# Novel paradigms to measure variability of behavior in early childhood: posture, gaze, and pupil dilation

**DOI:** 10.3389/fpsyg.2015.00858

**Published:** 2015-07-09

**Authors:** Robert Hepach, Amrisha Vaish, Michael Tomasello

**Affiliations:** ^1^Department of Developmental and Comparative Psychology, Max Planck Institute for Evolutionary AnthropologyLeipzig, Germany; ^2^Department of Psychology, University of VirginiaCharlottesville, VA, USA

**Keywords:** posture, eye tracking, Kinect, pupillometry, pupil dilation, emotion, children, internal arousal

## Abstract

A central challenge of investigating the underlying mechanisms of and the individual differences in young children’s behavior is the measurement of the internal physiological mechanism and the involved expressive emotions. Here, we illustrate two paradigms that assess concurrent indicators of both children’s social perception as well as their emotional expression. In one set of studies, children view situations while their eye movements are mapped onto a live scene. In these studies, children’s internal arousal is measured via changes in their pupil dilation by using eye tracking technology. In another set of studies, we measured children’s emotional expression via changes in their upper-body posture by using depth sensor imaging technology. Together, these paradigms can provide new insights into the internal mechanism and outward emotional expression involved in young children’s behavior.

## Introduction

Children’s navigation through the social world rests on a set of socio-cognitive abilities that emerge during infancy and enable children to tune in to others’ emotions and mental states (Carpenter et al., 1998; Tomasello et al., 2005; Grossmann and Johnson, 2007). These abilities in turn allow children to later establish and maintain social relationships. Nevertheless, there are individual differences with regards to the mechanisms that moderate children’s social navigation and there is variability in children’s responsiveness to others’ feelings and desires (Dunn et al., 1991; Eisenberg et al., 1996; Rothbart et al., 2000; Knafo et al., 2008; Salley et al., 2013).

A central challenge to measuring the underlying processes of behavior is that these processes are often either internal or partially based on expressive emotions that occur briefly and rapidly in succession. However, recent advances in eye tracking and depth sensor imaging technology (1) allow us to ‘listen in’ on the internal states underlying behavior, and (2) provide a new lens through which emotional expressions become visible. Here, we illustrate two novel paradigms recently developed in our lab: one on children’s responses to seeing others in need of help and another on children’s postural changes following goal-oriented behavior. In our studies, the technology captures children’s physiology and physiognomy from a distance so as to retain a natural setting in which the targeted behavior occurs.

The first paradigm was designed to capture children’s gaze and pupil dilation as indices of attention and changes in internal arousal, respectively, in behavioral studies. Both variables are frequently assessed in studies on infants’ physical and social cognition (Aslin and McMurray, 2004; Aslin, 2007; Falck-Ytter et al., 2013; Sirois and Brisson, 2014). However, while eye tracking has previously been employed to study cognition, our approach focuses on explaining children’s behavior and addressing questions concerning its underlying motives and proximate mechanisms in active behavioral paradigms with children. Here we illustrate the use of this method to address questions regarding the specific motivations underlying children’s prosocial behavior (see Gaze and Pupil Dilation). The second paradigm was designed to measure children’s emotions, in particular their positive emotions as expressed in their posture. Previous research had focused on facial expressions and composite measures of gestures and posture to identify positive affect in young children through human coders’ judgment. However, it has thus far not been possible to automatically capture children’s emotions and to focus on specific body parts, e.g., the chest and hip. We apply this technique in situations where children achieve an outcome for themselves and we measure how their posture accordingly changes compared to a prior baseline (see Posture). This allows us to address questions regarding the emotions that accompany behavior, e.g., positive affect following success.

Here we illustrate the two paradigms, which involve capturing children’s eye movements and pupil dilation to measure changes in autonomous nervous system (ANS) activity, and measuring children’s posture in order to assess changes in their emotional state. We propose that these measures can not only provide insights into the internal mechanisms and emotional bases of children’s behavior but also allow researchers to trace the physiological antecedents of children’s actions to better understand the sources of variability and individual differences in social cognition and behavior.

## Gaze and Pupil Dilation

For children to become competent social partners, they need to realize when others are in need of help, represent the appropriate solution, and have a sympathetic motivation to care for others’ needs (Eisenberg and Miller, 1987; Zahn-Waxler et al., 1992; Dunfield, 2014; Warneken, 2015). The attention children pay to others’ actions can be measured through tracking their eye movements and mapping them on a visual scene (Aslin and McMurray, 2004). Eye tracking is based on corneal reflection technology and provides numerous non-invasive indicators of attention, including fixations, saccades, anticipatory looking, and scan patterns (Aslin, 2007, 2012; Gredebäck et al., 2009; Oakes, 2012). This has opened up new ways of studying social cognition in infants and young children (Navab et al., 2012; Falck-Ytter et al., 2013; Tenenbaum et al., 2013). Both the time children spend looking at a scene and the pattern of eye movements can reveal the underlying structure of children’s social attention (Falck-Ytter et al., 2006; Aslin, 2007; Frank et al., 2012; Fawcett and Gredebäck, 2013; Elsner et al., 2014).

An additional feature of modern corneal reflection eye trackers is the automatic capture of pupil diameter (Wang, 2011). Similar to other physiological measures such as heart rate or skin conductance, changes in pupil dilation reflect activation of the ANS (e.g., Kahneman et al., 1969; Libby et al., 1973; Bradley et al., 2008). This is particularly interesting for developmental research because whereas eye movements can reflect the distribution of attention, changes in pupil dilation may provide a measure of the degree of psychological involvement in pre-verbal and just-verbal populations (see Goldwater, 1972; Laeng et al., 2012; Sirois and Brisson, 2014, for reviews). Similar to the measure of children’s eye movements, the measure of pupil dilation has also found application in infancy to study both physical and social cognition early in ontogeny (Falck-Ytter, 2008; Chatham et al., 2009; Jackson and Sirois, 2009; Gredebäck and Melinder, 2010, 2011; Geangu et al., 2011; Sirois and Jackson, 2012; Hepach and Westermann, 2013; Burkhouse et al., 2014).

The majority of previous work had implemented measures of gaze and pupil dilation in response to pictures or prerecorded video stimuli. To study more natural interactions researchers have adapted these set-ups for live paradigms wherein children sit facing an adult while the eye tracker records their eye movements and pupil size. Gredebäck et al. (2010) studied infants between the ages of 2–8 months in a live setting in which the experimenter or the mother sat facing the child. The authors found that infants’ ability to follow others’ gaze develops linearly and was more stable when facing a stranger compared to their mother (see Falck-Ytter et al., 2015, for a similar set-up). However, none of this previous work attempted to relate children’s eye movements and pupil dilation to their behavior as a way to capture the mechanisms underlying children’s behavior. Our aim was thus to extend the use of these measures in novel directions to study behavior more generally.

In one example we developed a paradigm to address questions regarding the motivation underlying young children’s prosocial behavior. Specifically, we investigated how changes in internal arousal relate to children’s own prosocial behavior. For this purpose we devised a behavioral helping paradigm within which we could capture children’s gaze and pupil dilation to investigate the mechanisms underlying young children’s helping behavior (Hepach et al., 2012). During the first 2 years of life children show a remarkable array of prosocial tendencies, including sharing with and comforting those in need of help (Zahn-Waxler et al., 1992; Warneken and Tomasello, 2009; Svetlova et al., 2010; Dunfield, 2014; Eisenberg and Spinrad, 2014; Paulus, 2014; Warneken, 2015). However, much less is known about children’s motives to help others. Therefore we inquired whether changes in young children’s internal arousal reflect their motivation.

**FIGURE 1:**
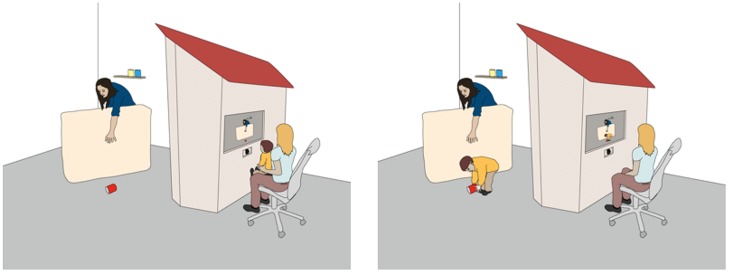
**The general set-up of the study.** While children sit in front of the computer monitor, the eye tracking unit records both their eye movements and pupil diameter before they return to the adult’s side.

The general set-up and procedure of our studies is comparable to other developmental studies on children’s prosocial behavior, yet we include a crucial difference. At pre-defined time points, children (24-month-olds) sit in front of an apparatus that resembles the facade of a house through which they can view the scene they themselves moments ago participated in. Children watch the scene on a computer screen, which shows a live video feed of the events on the ‘other’ side of the apparatus. Through a series of familiarizations (see also Troseth and DeLoache, 1998), children learn that what they see on the screen is actually happening and that they can return to the task they were engaged in before they sat down (see **Figure 1** for an illustration). In our studies, children view an adult carry out a task such as stacking cans until at one point the final object accidentally drops to the floor out of his/her reach. Children are then given the opportunity to subsequently help (see Hepach et al., 2012, for details).

**FIGURE 2:**
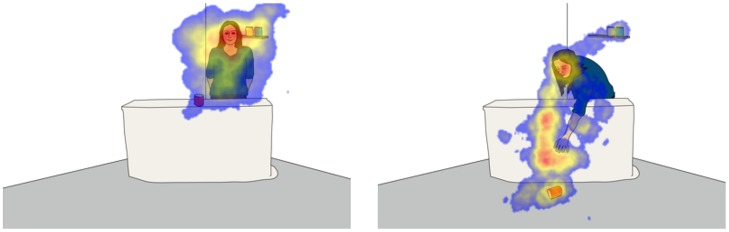
**Illustration of the scene children view while sitting in front of the apparatus.** In the actual studies, children view a camera image of the adult carrying out her task. Since her behavior is never identical across participants, the illustrations here represent prototypical poses during specific phases of the study. The **(left)** represents the time during which the adult carried out her task, e.g., stacking cans, standing behind the table. The **(right)** represents the time the adult spent reaching for the dropped object. Each phase can last from a few seconds to several minutes. During that time, the eye tracker records children’s gaze that can be mapped onto the illustrations to identify focal points of attention. The resulting focus maps reveal areas of low (blue) and high (red) attention.

While children sit in front of the apparatus, an eye tracker records both their eye movements and changes in pupil dilation (Tobii model X120, SMI models RED and RED-m). The live feed is presented on the computer screen by capturing a video from a USB webcam. The presentation software of the eye tracking system (Tobii Studio with Tobii systems and Experiment Center with SMI) allows both displaying a live video and simultaneously recording eye data at a frequency of at least 60 Hz and uses a standard calibration procedure to map children’s gaze onto the computer screen (Gredebäck et al., 2009). It is furthermore possible to apply the same *post-hoc* gaze correction techniques suggested for eye tracking experiments (Frank et al., 2012). This allows for children’s gaze to be mapped onto the live scene that they are observing and in turn provides a glimpse into the underlying process guiding their visual attention (see **Figure 2**). To further match children’s pupillary responses to the live scene, several additional steps are necessary.

Assessing changes in children’s internal arousal in active behavioral paradigms is particularly challenging given that pupil size variations are highly volatile in response to children’s body movement during the study. We therefore further developed a technique (first described in detail in Hepach et al., 2012), which focuses on a specific component of pupil dilation rather than a mean over a specified time window. Changes in pupil diameter are a function of both sympathetic and parasympathetic nervous system activity. This results in the typical pupillary oscillations both dilating and constricting the pupil. Even in the dark, the pupils constantly oscillate (Wilhelm, 1991), making the signal of pupil diameter changes over time highly volatile. These oscillations are very different from smooth sine wave patterns given that the magnitude of the positive peaks (dilation) and negative peaks (constriction) as well as the time interval between peaks varies (Loewenfeld, 1993). Psycho-sensory stimulation will increase pupil dilation such that the peaks are higher than before the presentation of the stimulus. Analyses of changes in pupil diameter focus on robust indicators of psychologically induced effects such as the number of oscillations over several minutes (Warga et al., 2009), peak dilation (e.g., Laeng et al., 2012) or the amplitude of the pupillary light reflex (PLR; Steinhauer et al., 2000).

The PLR is the characteristic shape of the change in pupil size upon the presentation of light. With increasing stimulus luminance the pupils constrict. Loewenstein (1920) studied the influence of various emotional states in clinical patients and observed the PLR to be inhibited during induced stress, e.g., when subjects experienced tension or witnessed a startling event. The elicitation of a PLR by shining a light into subjects’ eyes is part of the standard procedure in ophthalmology to assess parasympathetic and sympathetic nerves innervating both eyes (Wilhelm, 1991; see Bakes et al., 1990; Heller et al., 1990, for examples). Furthermore, several psychological stimuli inhibit the PLR, e.g., following negative emotional events (Bitsios et al., 2004) and with increased attention during task demands (Steinhauer et al., 2000). An increase in internal arousal results both in overall increased pupil dilation (Bradley et al., 2008) and in an inhibited PLR (Henderson et al., 2014; though see Nyström et al., 2015, for a different interpretation of the PLR in comparison to tonic pupil diameter). The advantage of measuring the PLR as an indicator of internal arousal is its quick assessment within 2–3 s. The crux is that the experimental manipulation has to occur before and not while the PLR is elicited.

**FIGURE 3:**
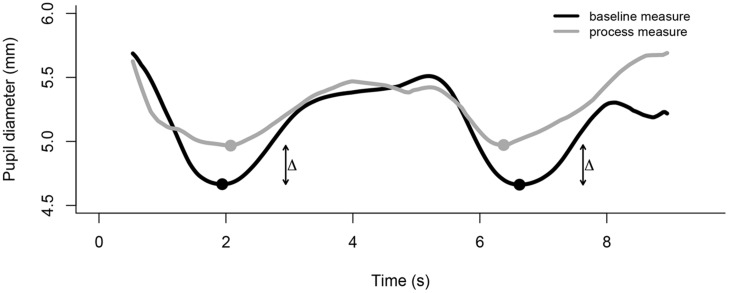
**The time course of the change in pupil size averaged across participants for two measurement time points, baseline and process.** For each measurement point two pupillary light reflexes (PLRs) are elicited through presenting a bright stimulus on a dark background at 0 and 5 s, respectively. Upon presentation of the bright stimulus, the pupils constrict and re-dilate after reaching their respective minimum. An algorithm identifies the two minima and calculates the change from baseline to process pupil diameter.

During behavioral studies the presentation of visual stimuli on a computer screen causes the pupils to constrict to the luminance properties of an image. We have developed a technique in which we elicit two PLRs in brief succession, i.e., a colorful image flashes twice on the computer screen. The recorded data are exported to a text file and processed using software such as R or Matlab. The exported data need to be pre-processed to remove extreme values (see Hepach et al., 2012, 2013, for filter and interpolation examples). Subsequently, an algorithm identifies the two pupil minima in response to the colorful image and averages both values. The raw value of pupil diameter, i.e., the average minimum, is reflective of individual differences in children’s arousal state. To further capture a change in children’s internal arousal in response to an experimental manipulation, we present the measurement image both before (baseline measure) and after (process measure) the experimental manipulation (e.g., seeing an adult needing help). The change is measured as the percentage increase from baseline to process (see **Figure 3**).

Through recording children’s eye movements as well as changes in pupil dilation in the domain of prosocial behavior, we have found that children’s own internal arousal increases when others are not helped but decreases to an equal degree when help is provided either by children themselves or by others (Hepach et al., 2012). Crucially, the degree of children’s internal arousal reflects individual differences in the latency with which they engage in helpful behavior, i.e., the greater children’s pupil dilation is after witnessing the situation, the faster they are to subsequently help others (see Hepach et al., 2013).

While gaze and changes in pupil dilation reveal the processes that precede and underlie a behavior, they do not provide information regarding valence, i.e., whether children’s responses are positive or negative in valence. For this purpose it is necessary to use an alternative measure of emotional expressiveness.

## Posture

Children’s attention to and involvement with others’ needs can explain the motivations leading up to a behavior. An equally important aspect of motivation is the question of how a behavior is maintained and reinforced. In the following, we illustrate a novel paradigm to measure the sorts of positive emotions that follow from successful behavior. From as early as 2 years of age, children show noticeable postural changes following their successful attainment of a goal. Such emotional expressions provide a window to assess a subject’s feelings, i.e., the internal state, especially if the emotion is studied in context (Lewis, 1997). Postural changes are accompanied by gestures such as pointing to the achievement or self-applauding (Heckhausen, 1987, 1988). By the age of 3, children display an erect posture after succeeding on difficult tasks and conversely their posture decreases if they fail on easy ones (Lewis et al., 1992). Adults display similar changes in posture when they feel proud (Shiota et al., 2003; Tracy and Robins, 2004; Horberg et al., 2013), following athletic success (Weisfeld and Beresford, 1982), as a cue of social dominance (Schwartz et al., 1982), social status (Shariff and Tracy, 2009), and expertise (Martens and Tracy, 2013).

Postural changes are reliably identifiable from a person’s gait (Montepare et al., 1987) as well as body movement (Dael et al., 2012) and the ability to detect pride from pictures emerges between 3 and 7 years of age (Tracy et al., 2005). The most common way to measure posture is to apply coding criteria to video recordings (e.g., Montepare et al., 1987; Heckhausen, 1988; Lewis et al., 1992; Shiota et al., 2003), photographs (e.g., Tracy and Robins, 2004; Shariff and Tracy, 2009; Martens and Tracy, 2013), drawings (Schwartz et al., 1982), and computer-animated mannequins or point light displays (Atkinson et al., 2004; Coulson, 2004). With these studies, then, there is a documented relation between success and the positive emotion expressed in an expanded upper-body posture. However, little research has assessed young children’s postural changes in naturalistic situations without relying on the coding of additional cues such as clapping (e.g., Heckhausen, 1987). Here, we introduce a recently developed paradigm using depth sensor imaging technology to automatically capture individual differences and changes in children’s posture.

### Automated Posture Assessment in Behavioral Paradigms

To track participants’ movement and the location of body points, we use a Mircosoft Kinect adapter in behavioral studies. The Kinect is a specialized camera that captures both an RGB-image, like any regular webcam, as well as the information of how far away each captured pixel is from the device itself. This depth sensor imaging is achieved through an emitted infrared light and a separate lens capturing the reflection of the projected light. If a person is within the device’s tracking range (∼1.5–4 m from the Kinect), the system estimates the *x, y*, and *z*-coordinates of up to 20 body points from the feet to the head (Shum et al., 2013; Stommel et al., 2015).

**FIGURE 4:**
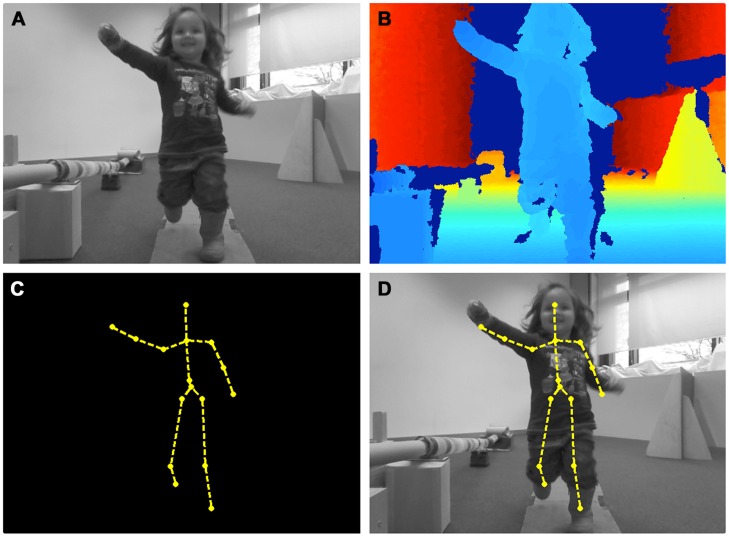
**The output of the Kinect motion sensor imaging technique. (A)** RGB-channel, **(B)** depth contour image, **(C)** estimated skeletal joints, **(D)** mapping of the skeletal joints onto the RGB image.

The Kinect allows for a relatively accurate and objective tracking of participants’ body points and an assessment of body posture expansion (see **Figure 4**). The technology has been employed in several contexts studying infant-caregiver interactions (Nagai et al., 2012), children’s behavior while playing cooperative games (Liu and LaFreniere, 2014), to assess and train motor abilities in clinical rehabilitation programs (Chang et al., 2013; de Greef et al., 2013; Luna-Oliva et al., 2013; Anzalone et al., 2014; Chung et al., 2014), and in interaction research (Won et al., 2014). In principal, the system can track multiple participants (Walczak et al., 2013) and it can be used to measure peripheral physiological measures such as respiratory rate (Burba et al., 2012). However, no experimental study has used the technology to capture the change in children’s body posture as an indicator of emotional expression. This was our aim in recent work, which we describe next. Before doing so, we present the results from a study with adults in an attempt to validate the use of the technology in this way.

### Validation Study with Adults

One of our central assumptions when using the Kinect technology in emotion research is that one can measure changes in upper-body posture that are related to positive and negative internal states. To test this assumption, we investigated whether the chest’s center is more elevated when adult participants experience a positive emotion compared to a negative emotion.

#### Participants

Twelve naïve adult subjects (6 female, 14 years 5 months to 37 years 10 months, median age 26 years 4 months) were recruited from the Max Planck Institute for Evolutionary Anthropology and gave informed consent prior to participating in the study.

#### Materials and Design

Each subject was asked to imagine experiencing four specific emotions, one at a time: joy, pride (positive emotions), disappointment, and guilt (negative emotions). We used a Kinect camera to record participants’ body posture, i.e., the height of their chest’s center as well as of their hip’s center. Adults were presented with four test trials with one emotion each. The order was counterbalanced across participants.

#### Procedure

Before the emotion trials, participants were asked to walk toward the Kinect camera (height = 0.85 m from the ground, distance from participants’ starting position = 3.7 m, angle of the camera = 11°) such that a baseline assessment of the position of the chest’s body joint could be made. The experimenter (blind to the study’s hypotheses) read out the following instructions to participants: “This is a validation for a method to measure emotional expression. For this purpose we would like participants to walk toward the Kinect twice. At the very beginning we will conduct a baseline measurement for which we ask you to walk in a relaxed natural manner. Afterward I will read out the instructions for each emotion, four in total.” At the beginning of each subsequent emotion trial the instructions were as follows (example joy): “The emotion to be displayed is joy. Can you recall an event that made you feel joy? Try to recall that feeling. Imagine the situation and surroundings. Take your time until you feel the emotion. Once you are ready give me a sign. Then you can walk toward the Kinect.” Next, participants walked in the direction of the Kinect while the positions of both the chest’s and hip’s body point were recorded. For each trial (including baseline) we asked participants to walk toward the Kinect twice to average the data from both movements.

#### Data Analysis

The data were recorded with and analyzed in Matlab (see details below, in Section “Studies with Children”). Data from one trial of one participant (disappointment emotion) could not be used for analyses because of a system failure during recording. For each participant we calculated the change in the chest’s height from baseline to the test trial for each of the four emotions and for each of 20 distance bins from the Kinect (1.2–3.2 m from the camera, 10 cm bin width). This controlled for differences in participants’ walking speed. Furthermore, we averaged the values of joy and pride as well as disappointment and guilt to arrive at one composite positive and one composite negative emotion change score. For statistical analyses we further binned the data into four time windows of equal length and computed Wilcoxon exact paired tests with Bonferroni correction for multiple testing (adjusted significance level *p*_adj_ = 0.0125). We carried out the identical analyses with children’s hip point height to investigate whether the effects of emotions were specific to the upper-body posture.

#### Results

**FIGURE 5:**
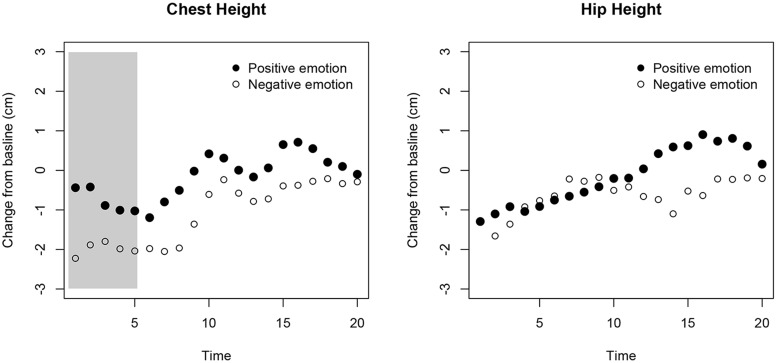
**Results from the adult validation study.** The *x*-axis represents the time after the emotion was elicited and as adults walked toward the Kinect. The *y*-axis shows the relative change in height for participants’ chest **(left)** and hip **(right)**. At each time point the median for the two positive and the two negative emotions is plotted. The gray area marks the time window where the difference between the two types of emotions was statistically significant (corrected for multiple testing).

Adults’ change in chest height from baseline was more elevated during the positive compared to the negative emotion events immediately after the emotion manipulation, *p* = 0.012 (all other *p*s > 0.1). This was not the case when performing the identical analyses on the hip’s center (all *p*s > 0.06; see **Figure 5**). These results suggested that adults’ upper-body varies with the valence of the induced emotion. Measuring changes in upper body posture using the Kinect system can tap into the types of internal states involved in the experience and display during emotional episodes. This potentially makes the technology an interesting research tool to assess emotional expressions in young children.

### Studies with Children

**FIGURE 6:**
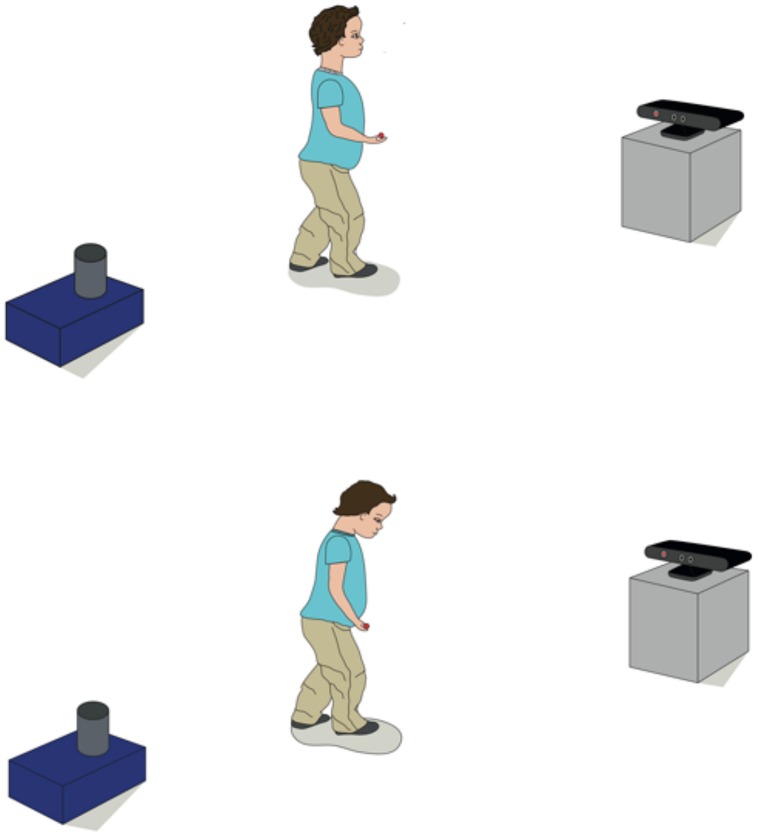
**The experimental setup of the study.** Children walked toward the Kinect camera after they either experienced a positive **(top)** or neutral event **(bottom)**. During the positive event children retrieved an object from the blue box that allowed them to continue an activity while in the neutral event no experimental manipulation occurred.

In our behavioral studies with 2-year-old children, participants can move around freely in a naturalistic setting without the need to attach point-light markers to their clothes. The Kinect ‘draws’ virtual points on participants’ bodies. At specific time points during the study, the child moves toward the Kinect camera so that a full body image can be captured. In the following we provide data from one example (not reported with the original study) to illustrate that children’s experience of an event that elicits a positive emotion reflects in changes of their body posture. At the beginning of the study we carried out a baseline measure during which children walked toward the Kinect without any experimental manipulation. At a later point in the study children manipulated a box to retrieve a toy that allowed them to continue with an attractive activity. Following this event, children again walked toward the Kinect camera. We hypothesized that experiencing this positive event would increase children’s upper-body posture (see **Figure 6** for an illustration).

The tracking of multiple body points allows one to isolate, for example, changes in shoulder and chest height from changes in hip height. That is, even though up to 20 body joints can be tracked with the Kinect, we focused on children’s upper-body posture following work on signs of pride in adults (Montepare et al., 1987; Tracy and Robins, 2004). More specifically, we calculated the height of the chest’s center as an indicator of postural expansion. Through assuming an upright posture, the shoulders are pushed back which in turn elevates the chest. In principle, a lowering of chest height could reflect a slumped posture, as documented in states of negative affect (Lewis et al., 1992).

**FIGURE 7:**
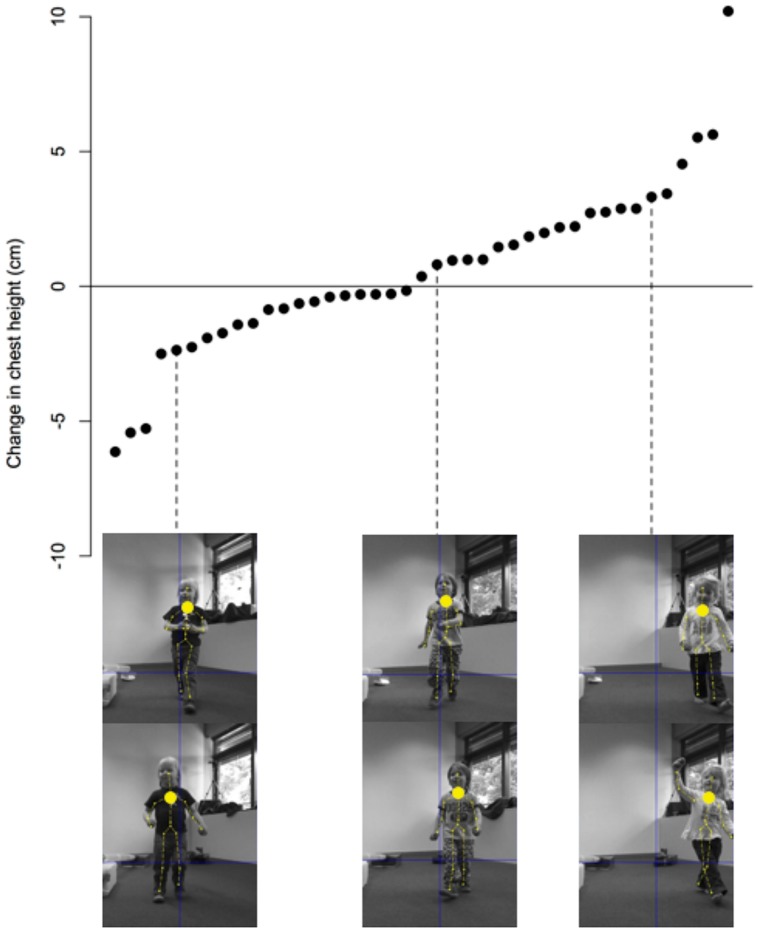
**An illustration of the data collected by the Kinect camera.** Each data point represents the change in the height of the chest’s center for each participant. The values are sorted from the lowest change to the largest change in height. For three participants illustrations are provided from the baseline sequence **(top row)** and from the later taken process sequence **(bottom row)**.

The data were recorded running a script written in Matlab. At regular time intervals, the program records (1) information regarding the position of each body point in three-dimensional space, (2) a color image, (3) a depth image, as well as (4) the location of each point on the two-dimensional color image (see **Figure 4**). Separate analyses (written partly in Matlab and R) calculate the difference in chest height between the baseline phase and the measurement taken during the test trial after the experimental manipulation. This results in a baseline-corrected change score that indicates the change in upper-body posture (see **Figure 7**).

#### Coding of Children’s Affect

The assumption underlying the interpretation of the Kinect data is that changes in children’s chest but not hip height reflect changes in positive affect. The more positive children feel, the more their upper-body posture should expand. To investigate this relation we asked two adults (blind to the study’s hypotheses and type of trial) to rate the recordings of children’s behavior along several dimensions.

The material consisted of the recordings of 48 children (25 girls, age range 29 months; 4 days to 31 months; 5 days; median age 30 months; 16 days) with two trials per child (baseline and test). The Kinect system could not record data for seven children on either trial or on both. For each trial coders were provided with the picture frames for the sequence when children started walking toward the Kinect, i.e., the exact same frames that were used for the automated posture analysis. The picture frames did not depict the skeletal information provided by the Kinect system.

For each trial the two coders were given the following instructions along with the SAM (self-assessment-manikin) rating scale (Bradley and Lang, 1994): “The SAM-rating consists of a valence coding and an arousal coding. The scale ranges from 9 to 1. For each trial, please answer the following questions: How pleasant is the emotion that the child is experiencing (very pleasant ∼9, very unpleasant ∼1)? How arousing is the emotion that the child is experiencing (very arousing ∼9, not at all arousing ∼1)?” In addition we asked coders what emotion they saw the child displaying and what features they paid attention to when identifying the emotion. The aim of the latter question was to investigate which emotions coders spontaneously associate with the behavior of the child. The ratings of the two coders were positively correlated, both with regards to rating valence [*ρ_spearman_*(*n* = 90) = 0.74, *p* < 0.001, ICC = 0.63] and arousal [*ρ_spearman_*(*n* = 90) = 0.42, *p* < 0.001, ICC = 0.36]. We therefore averaged both codings to arrive at composite measures of both valence and arousal.

**FIGURE 8:**
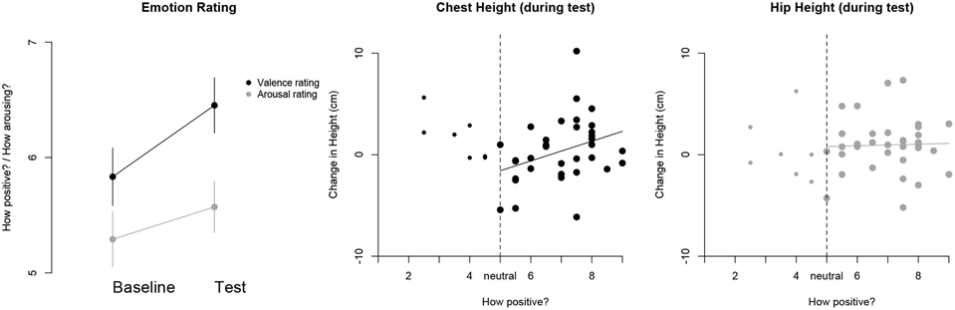
**(Left)** The average ratings regarding the valence and arousal of children’s expressed affect for the baseline and test trial, separately. The vertical bars represent standard error bars. **(Center)** The relation between the rated valence of children’s affect and their change in chest height for the test trial. The dashed vertical line represents the value corresponding to the neutral affect coding. Data points for the positive affect realm are highlighted and a regression line is added to illustrate the direction of the association. **(Right)** The relation between the rated change in children’s affect valence and the change in hip height for the test trial.

The results showed that the rated pleasantness of the children’s affect was greater in the test (*M* = 6.45, SD = 1.66) compared to the baseline (*M* = 5.83, SD = 1.73) trial, *t*(41) = 2.24, *p* = 0.031. On the other hand, there was no difference in the ratings of children’s arousal between the baseline and test trial, *t*(41) = 1.17, *p* = 0.25 (see **Figure 8**). This suggests that the experimental manipulation of attaining a goal for oneself makes children appear to experience more pleasure compared to a baseline level. With regards to the coders’ ratings of children’s affect during the test trial and the change in children’s posture, there was no overall relation between the two variables, *ρ*(*n* = 41) = 0.087, *p* = 0.59. However, very few trials were coded as ‘negative,’ i.e., with a value of less than 5 (17%). When focusing the analyses on the positive affect realm, i.e., ratings from 5 to 9, the degree of children’s experienced affect was positively related to the change in their chest height from the baseline to the test trial. Children with ratings of high positive affect also tended to show a greater increase in upper-body posture, *ρ*(*n* = 34) = 0.37, *p* = 0.03 (see **Figure 8**). On the other hand there was no such relation with respect to children’s lower-body posture, i.e., the change of hip height, *ρ*(*n* = 34) = 0.08, *p* = 0.67 (see **Figure 8**). In addition, no statistically significant relations emerged between children’s rated degree of arousal and the change in their chest or hip height, *p*s > 0.09. Furthermore, the most frequently rated emotion after the experimental manipulation was ‘happy’ (see **Table 1** for details) and the most frequent features that coders paid attention to were children’s smile, posture, and gait (see **Table 2** for details).

**Table 1:** The frequency of the type of emotion children expressed during the test trial as identified by the two coders.

	Angry	Sad	Embarrassed/uncertain	Disappointed	Disinterested	Surprise	Neutral	Active	Proud	Happy
Frequency	1	2	23	1	6	3	4	6	14	52

*For the 48 children each coder could list two possible emotions per trial. The initial codings were grouped into one of the 10 categories. The maximum possible frequency for an emotion was 96, i.e., both coders identified the emotion for each of the 48 trials*.

**Table 2:** The features of children’s behavior during the test trial that coders referred to when making the decision of what emotion the child was expressing.

	Smile	Other facial feature	Looking direction	Posture	Gait
Frequency	43	3	6	41	49

*Each coder could provide two features per trial. The initial codings were grouped into one of the five categories. The maximum possible frequency for a feature was 96, i.e., both coders referred to this feature for each of the 48 trials*.

Overall, the Kinect depth sensor imaging technology not only provides information on individual differences in overall body size but also registers subtle changes in posture. Given the link between positive affect and increased body posture, the Kinect is an extremely useful new tool to measure emotional expressions and motivational states in children.

## Summary and Future Directions

The paradigms described in this brief overview aim to capture the underlying mechanism of behavior and the types of expressive emotions that follow from it. Children’s eye movements in response to live events in behavioral paradigms reveal how they allocate their attention. Likewise, changes in their pupil dilation indicate the strength of their motivation. Individual differences in children’s internal arousal before they carry out an action are related to how quickly they do so. In addition to these measures of internal arousal, changes in upper-body posture reflect children’s positive emotional state after carrying out an action. A more straight and upright posture is indicative of a positive emotion while a hanging posture may reflect a negative emotional state (see also Lewis et al., 1992). These methods allow researchers to address novel questions regarding the underlying mechanisms of behavior (using pupil dilation) as well as children’s emotional expressions that accompany behavior (using depth sensor imaging). One direction for future studies using eye tracking systems is to collect gaze and pupil data without the need for children to look at a computer screen. The fact that children have to move out of the live situation and temporarily sit in front of a separate apparatus interrupts the task they are involved in. In particular, younger children have difficulty sitting on their parent’s lap after an engaging activity. This can result in inattentiveness and fewer chances to gather data points. Moreover, the image on the screen is only a 2-dimensional representation of a 3-dimensional space. In principle, both Tobii and SMI eye tracking systems can map participants’ gaze onto a ‘real’ scene. This is a direction for future research to explore, especially with the emergent use of head-mounted cameras and eye tracking systems (Aslin, 2009; Smith et al., 2014). With regard to using the Kinect camera, an interesting further step is to explore whether the system can also capture other emotions, including those with negative affect such as shame and guilt. In particular, investigating the relation between the various body points, e.g., head vs. shoulders, will provide an interesting avenue for future research given that we have thus far only explored the change in chest height. In this way the Kinect could not only be used to address questions regarding the positive emotions that follow from successful actions but also to study emotional expression in children more broadly.

In the examples given here, measures of pupil dilation assessed children’s internal arousal before they carried out an action while measures of posture were taken after children completed their action. In principle, neither technology needs to be restricted to these uses. In fact, there is now work measuring changes in internal arousal both before and after an experimental manipulation to investigate different motivations underlying children’s helping behavior (Hepach et al., 2012). Likewise, children’s emotional expression in their body posture may already change in anticipation of a positive or negative event.

The study of individual differences in children’s behavior relies in part on providing novel dependent measures with which to investigate subtle differences in behavior. In particular, studying the underlying mechanisms allows researchers to address questions that go beyond asking whether or not a behavior occurred in a given context. In the present paper we have provided the example of children’s prosocial behavior. Children, much like adults, do not help all the time and understanding the motivations that facilitate or inhibit helping is critical in our understanding of human prosociality. Changes in children’s internal arousal, as measured via variation in pupil dilation, do not only reveal how children respond to others in need but also systematically relate to their willingness to engage in helping. Children with greater pupil dilation in response to seeing a person in need are faster to subsequently help (Hepach et al., 2013). In more recent work we have found that children show greater pupil dilation when viewing an adult struggling with an instrumental task compared to a non-social case that portrays an instrumental problem without a person present (Hepach et al., in review). In that study we further specified that it is children’s process- but not baseline-measure that systematically relates to individual differences in helping behavior. Similar to the findings by Hepach et al. (2013), children with greater pupil dilation in response to seeing an adult in need were faster to help. Future work will have to investigate whether this relation between internal arousal and the latency to carry out a behavior is specific to children’s helping or whether it applies to other contexts as well (such as play).

In addition to understanding the underlying mechanisms of behavior it is equally important to study variability in the expressive emotions that accompany children’s behavior. Emotions can be measured via various modalities such as the voice or face. In the present paper we have illustrated a novel paradigm to measure children’s posture after achieving a positive outcome. Such changes are relevant to behavior given that children are more likely to carry out an action if they find it enjoyable. Children did not only show variability in their emotional expression (see **Figure 7**) but the change in their upper-body posture systematically related to adult coders’ ratings of the valence of children’s expressed emotion. Children with greater increase in posture were rated to feel more positive. One possible avenue for future research is to investigate how others perceive and respond to children’s postural changes which may in turn have an impact not only on how children’s subsequent posture changes but also on how they experience the actual emotion underlying the postural change (see also Carney et al., 2010).

Early in ontogeny, the technologies described here can provide a window into the underlying mechanisms of behavior. This is particularly relevant given that different processes can result in the same behavior (Karmiloff-Smith et al., 2014). The two examples in the present paper show that variability in children’s behavior is meaningful, e.g., children’s helping behavior is related to changes in their internal arousal. While the implementation of both pupillometry and depth sensor imaging was here illustrated in two specific contexts, their application need not be limited to children’s prosocial and goal-oriented behavior. In fact, any researcher interested in variability of behavior early in ontogeny may find the research tools illustrated here useful for various forms of behavior in different contexts. Internal measures of attention and arousal as well as measures of emotional expressiveness move researchers closer to the source of variability. Together, these measures can be considered additions to the scientific toolbox with which researchers study the origins and development of children’s social cognition and behavior. It will be a central challenge for future work to implement these techniques to study age-related changes in children’s social cognitive development.

## Conflict of Interest Statement

The authors declare that the research was conducted in the absence of any commercial or financial relationships that could be construed as a potential conflict of interest.
